# Data Interoperability of Whole Exome Sequencing (WES) Based Mutational Burden Estimates from Different Laboratories

**DOI:** 10.3390/ijms17050651

**Published:** 2016-04-29

**Authors:** Ping Qiu, Ling Pang, Gladys Arreaza, Maureen Maguire, Ken C. N. Chang, Matthew J. Marton, Diane Levitan

**Affiliations:** 1 Translational Molecular Biomarkers, Merck Research Laboratories, Merck & Co., Inc., 126 E. Lincoln Avenue, Rahway, NJ 07065, USA; ling.pang@merck.com (L.P.); gladys.arreaza@merck.com (G.A.); maureen.maguire@merck.com (M.M.); ken.chang@merck.com (K.C.N.C.); diane.levitan@merck.com (D.L.); 2 Companion Diagnostics, Translational Biomarkers, Merck Research Laboratories, Merck & Co., Inc., 126 E. Lincoln Avenue, Rahway, NJ 07065, USA; matthew_marton@merck.com

**Keywords:** next generation sequencing (NGS), whole exome sequencing (WES), mutational burden, immune checkpoint inhibitor, PD-1

## Abstract

Immune checkpoint inhibitors, which unleash a patient’s own T cells to kill tumors, are revolutionizing cancer treatment. Several independent studies suggest that higher non-synonymous mutational burden assessed by whole exome sequencing (WES) in tumors is associated with improved objective response, durable clinical benefit, and progression-free survival in immune checkpoint inhibitors treatment. Next-generation sequencing (NGS) is a promising technology being used in the clinic to direct patient treatment. Cancer genome WES poses a unique challenge due to tumor heterogeneity and sequencing artifacts introduced by formalin-fixed, paraffin-embedded (FFPE) tissue. In order to evaluate the data interoperability of WES data from different sources to survey tumor mutational landscape, we compared WES data of several tumor/normal matched samples from five commercial vendors. A large data discrepancy was observed from vendors’ self-reported data. Independent data analysis from vendors’ raw NGS data shows that whole exome sequencing data from qualified vendors can be combined and analyzed uniformly to derive comparable quantitative estimates of tumor mutational burden.

## 1. Introduction

### 1.1. Immune Checkpoint and Cancer Mutational Burden

In fighting cancer, no recent advance has been more promising than the rise of immune checkpoint inhibitor therapy. The first successes were in advanced melanoma [1], but subsequent evidence showed that immunotherapy works against a range of cancers [2]. Even for patients who have exhausted all standard of care treatments, the release from immune checkpoint blockade is able to halt cancer growth in a significant fraction of patients, most of the time with manageable and reversible adverse effects [3,4,5,6,7].

The US Food and Drug Administration (FDA) has approved three immune checkpoint monoclonal antibody therapies to date: ipilimumab targeting cytotoxic T-lymphocyte-associated protein 4 (CTLA-4); pembrolizumab and nivolumab targeting programmed cell death protein 1 (PD-1). Many recent studies have suggested that combining immune therapeutic agents from different classes of immune checkpoint inhibitors may provide even more benefit, although the combined regimens can be more toxic [8]. Although significant and durable response rates have been reported, clinical benefit of these treatments has been limited to a subset of patients and has not been observed in all tumor types [4,9], highlighting the need for predictive biomarkers to identify patients most likely to respond to treatment. Some of the best responses have been observed in melanoma and non-small-cell lung carcinoma (NSCLC), cancers largely caused by chronic exposure to mutagens such as ultraviolet light [10] and carcinogens in cigarette smoke [11], respectively. Several studies have demonstrated that higher somatic non-synonymous mutational burden assessed by whole exome sequencing (WES) is associated with clinical efficacy of anti-PD-1/anti-PD-L1 therapy [12,13,14]. A leading hypothesis behind this is that these tumors express a higher diversity of neoantigens that trigger an immune response. Hence, there is intense scientific interest to quantify and understand mechanisms leading to increased mutational burden, especially since mutational burden varies greatly within and across tumor types, ranging from 10 to 1000 s of mutations [15,16,17].

### 1.2. Current Status of Next Generation Sequencing (NGS) in Clinical Application

Next generation sequencing (NGS) technologies enable clinicians to make improved diagnostic and treatment decisions. Currently, however, only gene panel based NGS assays are regularly used for cancer subtype diagnosis using either fresh frozen or formalin-fixed, paraffin-embedded (FFPE) tissue [18]. Exome sequencing, which sequences the protein-coding region of the genome, has been rapidly applied to variant discovery in research settings, and recent increases in accuracy have enabled development of clinical exome sequencing for mutation identification and mutational burden estimation in cancer patients [12,19]. Routine clinical WES is still in its infancy and multiple challenges to widespread clinical WES implementation remain. First, the generation of high-quality WES data from archival FFPE tumor material remains challenging [20]. Second, clinical interpretation of WES data to direct patient treatment decisions in a way that leverages both clinical and biological knowledge is not trivial and is not standardized across laboratories. In addition, developing a method to interrogate plausibly actionable variants of uncertain significance remains challenging.

To make WES clinical implementation a reality, the platform must be robust and precise, enabling different laboratories to achieve similar results. To assess the current state of WES and data interoperability by commercial laboratories, we compared WES data and mutations reported by multiple vendors on the same set of DNA samples. The comparison was done at two levels: (1) mutations reported by the vendors; (2) unbiased and standardized data analysis and mutation comparison from the fastq data generated by the vendors on a common set of genes.

## 2. Interoperability of Whole Exome Sequencing (WES) Data from Different Labs

DNA was extracted from three pairs of tumor/normal matched samples using the Qiagen QiaAmp FFPE kit (Cat # 56404, Qiagen, Hilden, Germany). Two pairs were obtained commercially as patient FFPE blocks and one pair was a blended sample of two cell lines with a known mutational profile, which was used to assess WES assay sensitivity. The three tumor samples were denoted as S1, S2 and S3. Sample S1 is a mix of 30% DNA from tumor cell line HCC1143 and 70% DNA from normal cell line HCC1143BL derived from the same donor (fresh frozen DNA from ATCC, American Type Culture Collection) to mimic 30% tumor content. 178 non-synonymous mutations documented in the COSMIC (Catalogue Of Somatic Mutations In Cancer) database were expected to be observed in this sample and were used to estimate sensitivity of the WES from each vendor. Sample S2 is a FFPE tumor with matched normal from a Lynch syndrome donor in which high mutational burden is expected. Sample S3 is a FFPE tumor with matched normal from a microsatellite stable (MSS) lung cancer donor in which low mutation burden is expected. Five hundred nanograms DNA for each sample was sent to five vendors. WES was performed on all three sample pairs in duplicate at four vendors (Labs A, B, D, E). A single NGS run was done for the two FFPE tumor/normal pairs at Lab C. Sample S1 was not analyzed by Lab C.

### 2.1. Mutation Data Reported by Vendors

**Figure 1:**
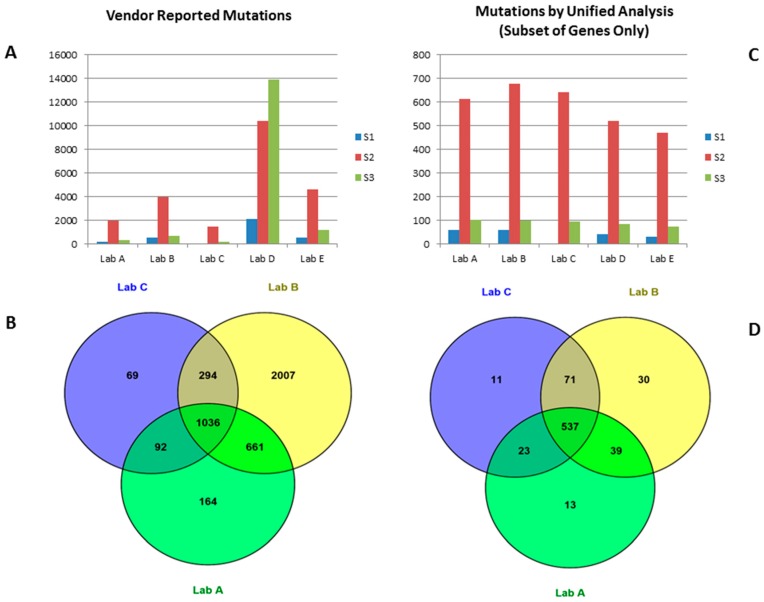
(**A**) Mutation number reported by all vendors for all three samples; (**B**) mutations reported by three top performing vendors (sample S2); (**C**) number of mutations detected by unified analysis of vendors’ fastq data. Only mutations in TruSight One gene panel regions were reported for all samples; (**D**) mutations detected by unified analysis of vendors’ fastq data. Only mutations in TruSight One gene panel regions were reported (sample S2).

All vendors used ~200 ng DNA as WES input. Vendors used a capture kit that was validated or optimized for their operation. The exome capture kits used include Agilent SureSelect v4, v5 (Agilent Technologies, Santa Clara, CA, USA), Illumina’s Rapid Capture exome kit (Illumina, San Diego, CA, USA) and one proprietary kit. Each vendor delivered on average ~100× on target coverage (Table 1). Somatic mutations were detected and reported by each vendor using their internal bioinformatics pipeline. The somatic mutations were then extracted from vendor mutation reports (vcf, excel or txt files) and integrated for comparison. A unique mutation was defined by a genomic coordinate as well as the base-pair change. Mutation detection concordance of the replicates was calculated by the number of shared mutations among the two replicates divided by the average of the number of mutations reported by the two replicates. The concordance of mutations detected from the two replicates varied from 3% to 89% for different vendors (Table 1). When a set of known 178 COSMIC mutations in S1 were evaluated for sensitivity from different vendors, vendor B identified 160 mutations for a sensitivity of 90%, while vendor D only achieved 55% sensitivity (Table 2). Given that the tumor cell line only accounts for 30% of the mix, the expected “somatic mutation” allele frequency is 15% (heterozygous mutation) or 30% (homozygous mutation). It is not surprising that WES will miss some low (*i.e.*, 15%) variant allele frequency (VAF) mutations due to the low and non-uniform coverage of a typical WES experiment (average ~100× in this study). Vendors reported a wide range in terms of number of mutations for the microsatellite stable sample S3 (from ~180 to ~23,400), indicating the impact of different variant calling pipelines and different filtering stringency applied. It is important to note that the number of reported mutations from different vendors varied drastically (Figure 1A); in one case the number of mutations called differed by 80-fold (vendor A and vendor D for the same sample S3, Table 1). This indicates that comparison of mutational burden data derived from WES by different vendors is very risky and could easily lead to contradictory results (hypermutator *vs.* hypomutator) unless the vendor process (data generation and data analysis) has undergone rigorous analytical validation and has been standardized with respect to the bioinformatics pipeline. It is also interesting to note that higher coverage did not automatically translate to high sensitivity, for example, Lab B achieved the best sensitivity on sample S1 while its coverage was not the highest.

### 2.2. Mutation Calls from Vendors’ Raw Data Using Unified Pipeline

Figure 1B shows that mutations reported by different vendors share a common set of mutations, however each vendor has a set of private mutations that were uniquely identified. For clarity, only the three top performing labs’ data are shown. The top performing labs were selected based on the number of detected mutations shared by other labs (data not shown). Many factors likely contributed to the large discrepancy among data reported by different vendors: Different capture kit with different bait size, bioinformatics pipeline, mutation call cutoff, filtering mechanism to remove germline mutations and sequencing artifacts, to name a few. In order to mitigate the impact of those factors, we performed sequence analysis and somatic mutation calling from the vendors’ raw data (fastq files) using The Genome Analysis Toolkit (GATK)/Mutect-based internal pipeline. Fastq files were provided by each vendor or regenerated from the vendor’s bam files. Reads mapping and variant calling was performed using each respective capture kit’s targeted region (bed file). Due to different capture kits used by the vendors, to ensure mutations from the same genomic regions were compared, we restricted our comparison to the genomic region that contained the 4813 genes included in the Illumina TruSight One gene panel (Illumina, San Diego, CA, USA), based on the assumption that the disease related gene regions were optimized by each capture kit. Table 1 (right half) shows the number of somatic mutations reported using the same pipeline for all vendors’ data within these common genes. In contrast to the comparison of vendors’ self-reported mutations, the number of mutations identified by the same pipeline is much more comparable with very few private mutations from each vendor (Figure 1C,D). For example, for sample S2 (Lynch syndrome sample), ~600 somatic mutations were detected from Vendor A, B, and C’s raw data set while ~100 mutations were detected for sample S3 (Microsatellite Stable (MSS) sample) (Figure 1C). Table 1 also shows that the mutations detected by all vendors have much improved concordance. Although each vendor still has a small number of private mutations, manual inspection suggests this is likely due to the allele frequency falling below the arbitrary chosen cutoff during variant calling in one vendor, but not the other. The data from vendor D and vendor E seems to have poorer quality comparing with other three vendors based on their low sensitivity to detect known mutations in S1 and the large number of reported mutations not reported by any other labs. Nevertheless, our study demonstrated that underlying data quality from high performing vendors is very comparable despite using different capture kits. Thus, it appears that whole exome sequencing data from quality vendors can be combined and analyzed uniformly to derive comparable quantitative estimate of mutational burden. It is important to stress that data are likely not comparable if the vendor mutation reports are used directly, *i.e.*, without the application of a single data analysis pipeline.

## 3. Discussion

Recent studies by Campesato *et al.* (2015) [21] and Rosenberg *et al.* (2016) [14] demonstrated that comprehensive cancer-gene panels can be used to estimate mutational burden and predict clinical benefit to PD-1 blockade in clinical practice. However, WES is still the preferred platform due to the fact that many of the neoantigens that are needed for future immune therapy exploration are outside the common gene panels. Without a doubt, WES remains to be a challenging assay for FFPE samples; in addition, a matched normal is highly recommended for interpreting somatic mutations [22]. As the data from the cell line mix sample (S1) shows that tumor content is another key factor in determining the value of WES mutational burden data. A typical sequencing depth for WES is around 100× which means only mutations with allele frequency >15% can be detected with confidence. This translates to a minimum of 30% tumor content required for the WES assay (30% VAF for homozygous somatic mutations and 15% VAF for heterozygous somatic mutations). Pre-analytical microdissection of FFPE slides is strongly recommended for low tumor content samples to increase tumor fraction and thereby to ensure accurate mutational burden estimate using WES.

## 4. Conclusions

In summary, our study suggests that in clinical practice, caution needs to be taken when comparing mutational burden data from different laboratories. This likely also applies to mutational burden estimates using the large NGS panels approach. To ensure data interoperability of tumor somatic WES data from different sources, the ideal solution may be for the NGS community to define a common framework in which experimental and data analysis parameters are documented and standardized and to establish a data commons at which raw data from different platforms can be shared. The critical steps to control or document include tumor content estimation, FFPE DNA isolation, input DNA quantification, DNA quality (amplifiability) determination, capture kit, NGS run quality, algorithms applied and parameter setting for reads assembly, variant calling, germline variant filtering, FFPE and genomic background specific artifact removal, *etc.* Our data show that in the interim a unified data analysis (controlled pipeline, identical filtering mechanism, same genome regions, *etc.*) is necessary in order to use commercial or local WES assays to derive mutational burden information and make clinical decisions on patients across multiple sites with pre-defined cutoffs. This can be achieved through centralized bioinformatics analysis or through a distributed cloud based analysis pipeline. Direct comparison of mutational burden data from different sources with different analysis pipelines might be misleading and contradictory, and may not deliver the most benefit to the patient.

## Figures and Tables

**Table 1:** Whole exome sequencing (WES) performance for all 5 vendors. Concordance is calculated by the number of shared mutations among the two replicates divided by the average of the number of mutations reported by the two replicates. Sample S1 data is not available for Lab C (as N.A. in table).

Vendor	Sample	Coverage	Mutations Reported by Each Vendor				Mutations Detected by Unified Analysis (for Genes in TruSight One Only)			
			Replicate 1	Replicate 2	Overlap	Concordance	Replicate 1	Replicate 2	Overlap	Concordance
Lab A	S1	181	200	200	178	89	60	62	54	88.52
Lab B	S1	135	519	553	391	72.95	61	61	57	93.44
Lab C	S1	N.A.								
Lab D	S1	103	2113	2144	367	17.24	42	41	33	79.52
Lab E	S1	66	510	622	327	57.77	29	38	25	74.63
Lab A	S2	144	1953	1949	1634	83.75	612	627	555	89.59
Lab B	S2	119	3998	3991	3470	86.87	677	656	623	93.47
Lab C	S2	170	1491	N.A.	N.A.	N.A.	642	N.A.	N.A.	N.A.
Lab D	S2	78	10,428	17,944	1968	13.87	519	573	466	85.35
Lab E	S2	82	4619	5201	3788	77.15	472	568	442	85
Lab A	S3	242	336	338	296	87.83	104	110	97	90.65
Lab B	S3	133	871	823	611	72.14	98	103	90	89.55
Lab C	S3	242	187	N.A.	N.A.	N.A.	94	N.A.	N.A.	N.A.
Lab D	S3	67	13,892	23,410	573	3.07	84	90	77	88.51
Lab E	S3	80	1214	1255	699	56.62	72	84	64	82.05

**Table 2:** WES accuracy estimate using tumor and normal cell line mix (sample S1). Thirty percent DNA from tumor cell line HCC1143 and 70% DNA from normal cell line HCC1143BL derived from same donor. Total 178 COSMIC mutations were expected. Sample S1 data is not available for Lab C (as N.A in the table).

Lab	Capture Kit Used	Total SNP Detected		Number of SNP Detected in COSMIC (Sensitivity of Detection in %)	
		Replicate 1	Replicate 2	Replicate 1	Replicate 2
Lab A	Illumina Rapid Capture Exome Kit	200	200	142 (80%)	141 (79%)
Lab B	Proprietary Kit	519	553	161 (90%)	159 (89%)
Lab C	Agilent SureSelect v4	N.A.			
Lab D	Agilent SureSelect v4	2113	2144	104 (58%)	98 (55%)
Lab E	Agilent SureSelect v5 + UTR	510	622	116 (65%)	142 (80%)
